# An Uncommon Expression of Immunoglobulin G4 (IgG4)-Related Disease: Sclerosing Mesenteritis Concomitant With IgG4-Related Autoimmune Pancreatitis

**DOI:** 10.7759/cureus.50529

**Published:** 2023-12-14

**Authors:** Ilias Bennouna, Maria Antonietta Bali, Maria Gomez Galdon, Ana Veron Sanchez

**Affiliations:** 1 Radiology, Centre Hospitalier Interrégional Edith Cavell (CHIREC) Braine l'Alleud, Bruxelles, BEL; 2 Radiology, Institut Jules Bordet, Bruxelles, BEL; 3 Radiology, Hôpital Universitaire de Bruxelles, Institut Jules Bordet, Brussels, BEL; 4 Pathology, Hôpital Universitaire de Bruxelles, Institut Jules Bordet, Brussels, BEL

**Keywords:** igg4 related pseudotumor, sclerosis mesenter, mesenteritis, igg4-related pancreatitis, igg4 disease

## Abstract

A 63-year-old male presented to our oncological hospital with a one-year evolving abdominal pain, with an abdominal mass feeling. Contrast-enhanced computed tomography displayed two soft tissue masses, one at the mesentery root and the second around the pancreatic tail; at the same time the patient presented with hyperlipasemia. Endoscopic biopsy for the pancreatic mass and surgical biopsy of the mesenteric one were performed in order to narrow diagnosis. No neoplastic cells but only dense fibro-inflammatory changes with immunoglobulin G4 (IgG4)-positive plasma cell inclusions were observed for both biopsies. A diagnostic and therapeutic strategy based on high suspicion of IgG4-related disease was adopted, with good clinical and imaging response to corticotherapy.

## Introduction

Immunoglobulin G4-related disease (IgG4-RD) is a rare immune-mediated fibro-inflammatory condition with a wide range of organs involved [1,2]. Progressive destruction of involved structures results from a growing deposition of IgG4-positive plasma cells within their tissues, leading to chronic inflammation and fibrosis [1]. Even though physio-pathogenic and immunological mechanisms behind this disease are yet to be totally understood, histopathological patterns are well established and very similar through all the organs possibly involved [3]. The mesenteric manifestation of IgG4-RD referred to as immunoglobulin G4-related sclerosing mesenteritis (IgG4-RSM) is an uncommon expression of IgG4-RD.

## Case presentation

A 63-year-old Caucasian male patient presented to our oncological hospital with a one-year evolving abdominal pain radiating to the back, with an abdominal mass feeling and a recent constipation. The physician only palpated epigastric mass with no peritoneal irritation symptoms at physical examination. The patient had a medical history of gastroduodenal peptic ulcers, a collarbone fracture and a family oncological history (sister breast cancer at 35 years old and father lung cancer at 58 years old). On laboratory examination complete blood count, cytolysis hepatic markers, renal and hepatic function, blood coagulation, ionogram, serum IgG4 concentration and tumor markers were normal. However, lipasemia was at 363 UI/L, above the normal range (60-260 UI/L).

**Figure 1:**
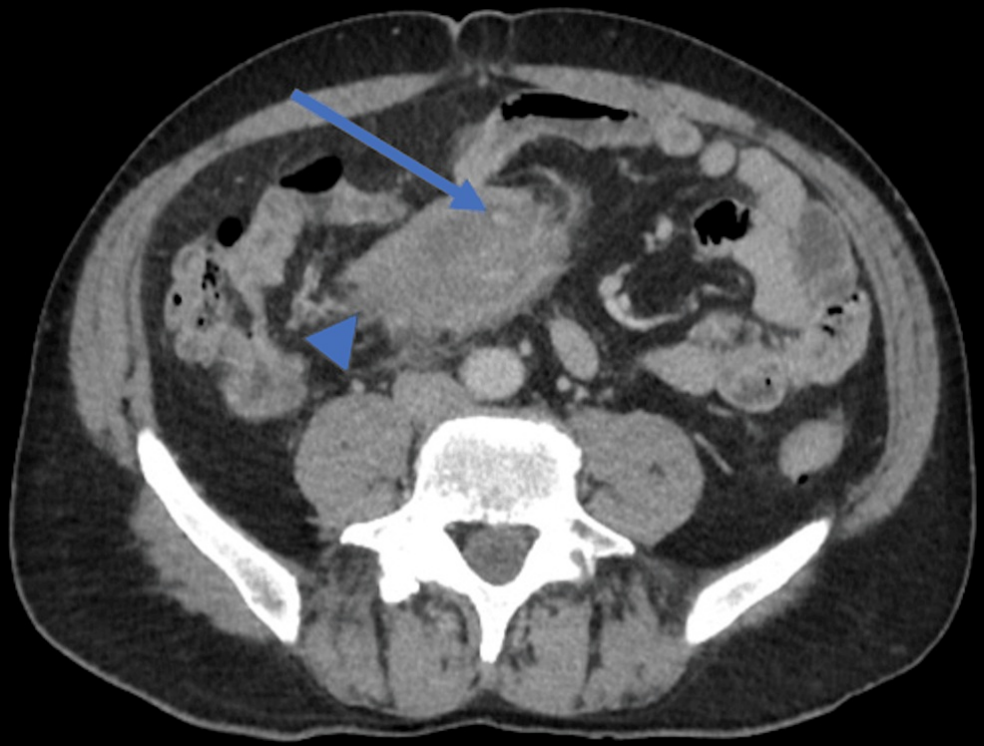
Initial contrast-enhanced computed tomography (CECT) CECT soft mesenteric root mass (arrowhead) displaying a small amount of preserved fat around a mesenteric vessel called the halo sign (arrow).

**Figure 2:**
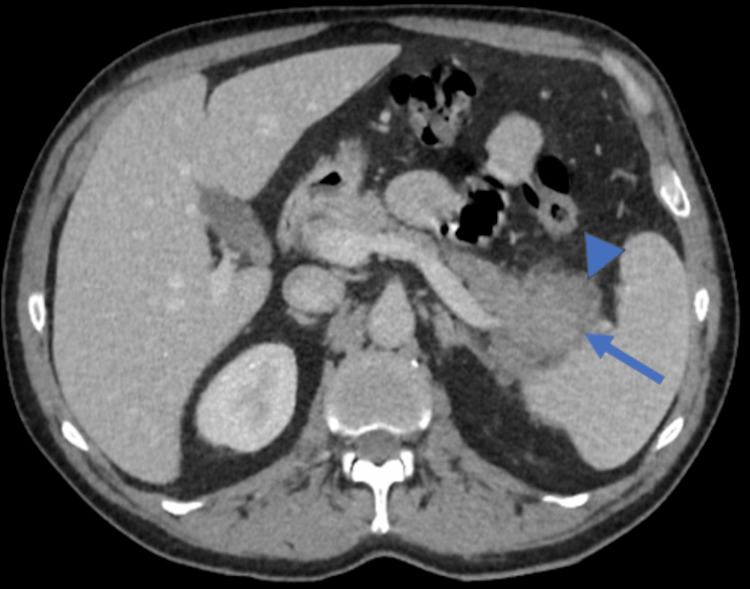
Initial contrast-enhanced computed tomography (CECT) Soft mass around the pancreatic tail (arrowhead). We can notice the moderate enlarged pancreatic tail within the mass, compared to its body (arrow).

Contrast-enhanced abdominal computed tomography displayed a mesentery root soft mass with no noticeable mass effect on surrounding structures, with a small amount of fat preserved around some mesenteric vessels (Figure 1). A second mass around the pancreatic tail was observed with at the same time an enlargement of the pancreatic tail (Figure 2). A small amount of ascites was noticed in the pelvic floor.

**Figure 3:**
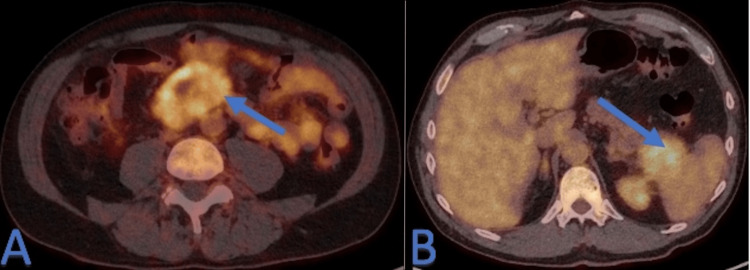
Initial F-fluorodeoxyglucose (FDG) positron emission tomography/computed tomography (PET-CT) We can observe the FDG uptake on both mesenteric (arrow) on A and pancreatic mass (arrow) on B.

Positron emission tomography/computed tomography (PET-CT) displayed a low to moderate F-fluorodeoxyglucose (FDG) uptake of both masses and the pancreatic tail (Figure 3).

First an echo-endoscopic biopsy of the pancreas and the mass around the pancreatic tail, then a surgical biopsy of the mesenteric mass was performed to narrow the differential diagnosis between a neoplastic cause, more likely a lymphoma due to the soft tissue nature of the different masses and an IgG4-RD with the concomitance of an IgG4-RSM and an IgG4-related autoimmune pancreatitis (IgG4-RAP).

**Figure 4:**
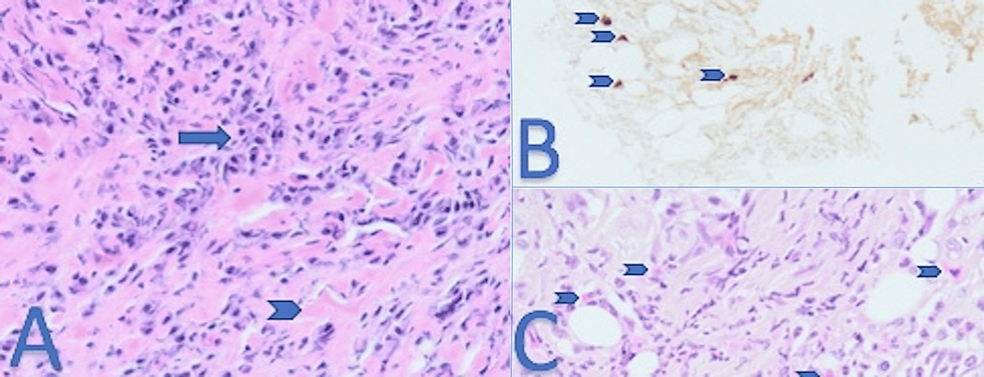
Pathology (A) The mesenteric tissue specimen reveals storiform fibrous proliferation (arrowhead) infiltrated by plasma cells and lymphocytes (arrow). (B) Immunostaining of IgG4-positive plasma cells (arrowhead). (C) Scattered eosinophils (arrowhead).

Histopathology of both biopsy samples showed storiform fibrosis, no obliterative phlebitis but a substantial white cell infiltration of fat tissues (lymphocytes, plasmocytes and neutrophils), some of the plasma cells were IgG4-positive (Figure 4), and no neoplastic cells were found.

**Figure 5:**
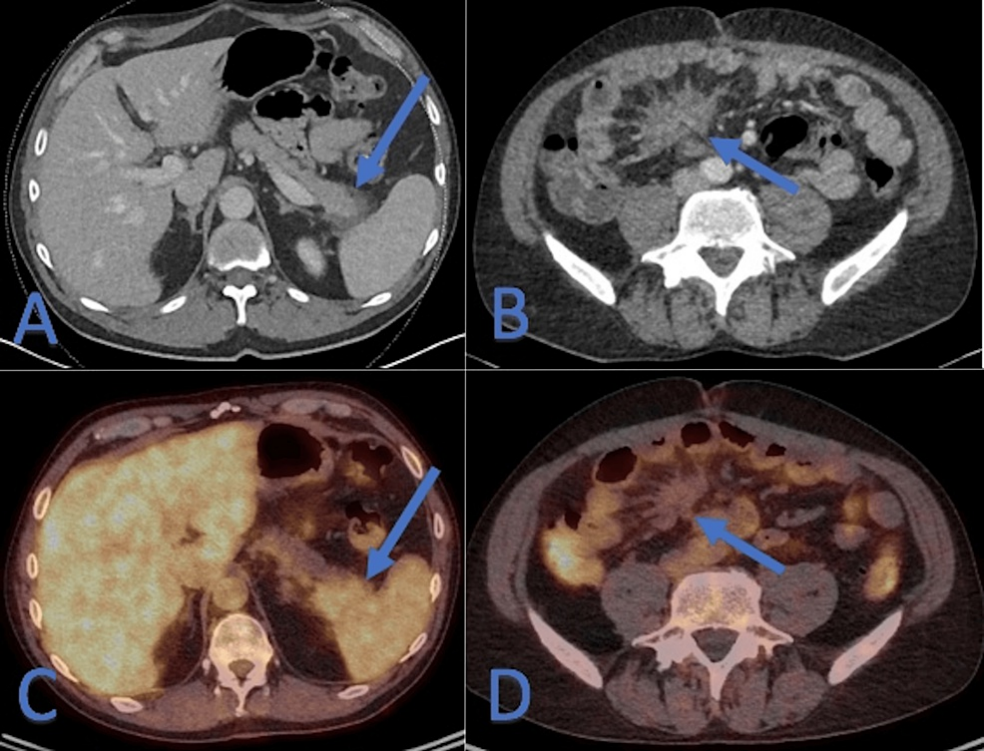
Six-week follow-up Contrast-enhanced computed tomography (CECT) (A and B) and F-fluorodeoxyglucose (FDG) positron emission tomography/computed tomography (PET-CT) (C and D) after six weeks of treatment. Size and activity decrease of the pancreatic and mesenteric masses (arrows in A, B, C and D); notice the recovery of the normal aspect of the pancreatic tail (arrow on A).

Based on the radiological biological and histopathological findings, the multidisciplinary medical team suspected IgG4-RSM with a concomitant IgG4-RAP and opted for a prednisone test and treat strategy. Prior to corticotherapy, IgG4 serum level was at 33.6 mg/dl (normal) and quiescent tuberculosis was assessed with a low dose Chest CT and Quantiferon tuberculosis (TB) serum level. After two weeks of oral prednisone at 0.6 mg/kg per day, patient's symptoms decreased. An abdominal CECT and PET-CT were performed at six weeks and at 18 weeks of treatment showed a size and activity decrease of the mesenteric and peri-pancreatic pseudo-masses with recovery of the normal aspect of the pancreatic tail (Figure 5). Prednisone was tapered after six weeks of treatment with a resolution of symptoms in the third month.

## Discussion

Sclerosing mesenteritis (SM) is a rare idiopathic inflammatory disorder with varying degrees of inflammation, fat necrosis and fibrosis [4]. IgG4-RD is a recently studied clinical entity, a systemic disease with a wide range of organs involved, including the mesentery and retroperitoneum. It consists of chronic inflammation with infiltration of IgG4-positive plasma cells within tissues involved. IgG4-RSM is an entity that started to emerge in the literature at the beginning of the 2010s, but remains sparsely reported [5-9].

Diagnosis criteria rely on clinical features, IgG4 serum levels, IgG4-positive plasma cells and pathological features such as storiform fibrosis, obliterative phlebitis and eosinophilia [10,11]. In our case, diagnosis was not certain after both biopsies; there was storiform fibrosis but no obliterative phlebitis, few IgG4-positive plasma cells and eosinophilia. IgG4 serum levels before treatment were not elevated. However, the multi-organ involvement because of the systemic nature of this disease, even though all imaging characteristics of an IgG4-RAP were not met [12], and the fact that the patient responded clinically and at imaging to corticotherapy, were strong arguments for IG4-RD diagnosis. Indeed glucocorticoid responsiveness is considered as a major diagnosis criterion [13]. Additionally, radiological features of the mesenteric lesion were in line with an IgG4-RSM, displaying features such as soft mass appearance and fat preserved ring around mesenteric vessels. We can then claim with a high level of confidence that our multidisciplinary team was facing an IgG4-RSM case with a concomitant IgG4-RAP.

Our patient had chronic abdominal pain and an abdominal mass feeling. Patients with IgG4-RSM can present diverse symptoms from digestive symptoms such as chronic abdominal pain, abdominal distention, abdominal mass feeling, nausea, vomiting and diarrhea to more systemic symptoms such as fever, anorexia, and weight loss [8].

Usually, diagnosis is made after explorative surgery with pathological examination due to the fact that IgG4-RSM mimic neoplastic masses [9].

The recommended initial systemic treatment for abdominal IgG4-RD is glucocorticoids [13]. Immunosuppressive agents can be considered after relapse or to maintain remission. More studies should be conducted to specify treatment modalities and treatment recommendations for IgG4-RSM that is a proper entity. Optimal treatment and prognosis may vary across different abdominal expressions of IgG4-RD.

## Conclusions

Our case displayed an IgG4-related sclerosing mesenteritis with concomitant IgG4-related autoimmune pancreatitis. The multi-organ involvement, radiological, biological, histopathological findings and glucocorticoid responsiveness favored an IgG4-related disease, even though pathological diagnosis elements were not met to assert a definitive diagnosis.

## References

[1] Umehara H, Okazaki K, Masaki Y (2012). A novel clinical entity, IgG4-related disease (IgG4RD): general concept and details. Mod Rheumatol.

[2] Della-Torre E, Lanzillotta M, Doglioni C (2015). Immunology of IgG4-related disease. Clin Exp Immunol.

[3] Cheuk W, Chan JK (2010). IgG4-related sclerosing disease: a critical appraisal of an evolving clinicopathologic entity. Adv Anat Pathol.

[4] Danford CJ, Lin SC, Wolf JL (2019). Sclerosing mesenteritis. Am J Gastroenterol.

[5] Chen TS, Montgomery EA (2008). Are tumefactive lesions classified as sclerosing mesenteritis a subset of IgG4-related sclerosing disorders?. J Clin Pathol.

[6] Minato H, Shimizu J, Arano Y, Saito K, Masunaga T, Sakashita T, Nojima T (2012). IgG4-related sclerosing mesenteritis: a rare mesenteric disease of unknown etiology. Pathol Int.

[7] Mori E, Kamisawa T, Tabata T (2015). A case of IgG4-related mesenteritis. Clin J Gastroenterol.

[8] Butt Z, Alam SH, Semeniuk O, Singh S, Chhabra GS, Tan IJ (2018). A case of IgG4-related sclerosing mesenteritis. Cureus.

[9] Bertoni M, Giani A, Tozzini S, Di Natale ME (2022). Sclerosing mesenteritis as an uncommon site of involvement of IgG4-related disease: a case report with an updated review of the literature. Cureus.

[10] Umehara H, Okazaki K, Nakamura T (2017). Current approach to the diagnosis of IgG4-related disease - combination of comprehensive diagnostic and organ-specific criteria. Mod Rheumatol.

[11] Deshpande V, Zen Y, Chan JK (2012). Consensus statement on the pathology of IgG4-related disease. Mod Pathol.

[12] Martínez-de-Alegría A, Baleato-González S, García-Figueiras R, Bermúdez-Naveira A, Abdulkader-Nallib I, Díaz-Peromingo JA, Villalba-Martín C (2015). IgG4-related disease from head to toe. Radiographics.

[13] Löhr JM, Beuers U, Vujasinovic M (2020). European Guideline on IgG4-related digestive disease - UEG and SGF evidence-based recommendations. United European Gastroenterol J.

